# Incretin-Based Therapies and Cancer: What’s New?

**DOI:** 10.3390/medicina61040678

**Published:** 2025-04-07

**Authors:** Sanja Medenica, Jelena Bogdanovic, Jelena Vekic, Tanja Vojinovic, Ivana Babic, Ljiljana Bogdanović, Viviana Maggio, Mohamed El Tanani, Manfredi Rizzo

**Affiliations:** 1 Department of Endocrinology, Internal Medicine Clinic, Clinical Centre of Montenegro, 81000 Podgorica, Montenegro; 2 Faculty of Medicine, University of Montenegro, 81000 Podgorica, Montenegro; 3 Clinic for Endocrinology, Diabetes and Metabolic Diseases, University Clinical Centre of Serbia, 11000 Belgrade, Serbia; jeca.bogdanovic@yahoo.com; 4 Faculty of Medicine, University of Belgrade, 11000 Belgrade, Serbia; bogdanoviclj77@gmail.com; 5 Department of Medical Biochemistry, Faculty of Pharmacy, University of Belgrade, 11000 Belgrade, Serbia; jelena.vekic@pharmacy.bg.ac.rs; 6 Study Program Pharmacy, Faculty of Medicine, University of Montenegro, 81000 Podgorica, Montenegro; tanjavojinovic88@gmail.com; 7 Emergency Center, University Clinical Center of Serbia, 11000 Belgrade, Serbia; babicivana993@gmail.com; 8 Institute of Pathology, Faculty of Medicine, University of Belgrade, 11000 Belgrade, Serbia; 9 School of Medicine, PROMISE Department of Health Promotion Sciences Maternal and Infantile Care, Internal Medicine and Medicinal Specialties, University of Palermo, 90133 Palermo, Italy; viviana.maggio01@unipa.it (V.M.); manfredi.rizzo@unipa.it (M.R.); 10 College of Pharmacy, Ras Al Khaimah Medical and Health Sciences University, Ras Al Khaimah P.O. Box 11127, United Arab Emirates; eltanani@rakmhsu.ac.ae

**Keywords:** DPP-4 inhibitors, GLP-1/GIP agonists, diabetes mellitus, obesity, cancer

## Abstract

Growing interest in incretin-based therapies for diabetes mellitus has led to an increased evaluation of their potential effects on cancer development. This review aims to synthesize recent evidence regarding the relationship between incretin-based therapies and cancer risk. We conducted a comprehensive literature review focusing on studies investigating dipeptidyl peptidase-4 (DPP-4) inhibitors, glucagon-like peptide-1 (GLP-1) receptor agonists, and dual GLP-1/glucose-dependent insulinotropic polypeptide (GIP) receptor agonists in relation to various malignancies. Current findings suggest that while these therapies demonstrate potential benefits, including weight reduction and metabolic regulation, concerns remain regarding their long-term safety profile. Notably, some studies indicate an increased risk of thyroid and pancreatic cancers, while others report protective effects against prostate, colorectal, and breast cancers. Given the complexity of their effects, further long-term studies and post-marketing surveillance are warranted. This review highlights the need for careful clinical assessment when prescribing incretin-based therapies to patients who may be at increased risk of cancer.

## 1. Introduction

Type 2 diabetes mellitus (T2D), a lifelong condition, poses significant therapeutic challenges due to its multifaceted pathophysiology and associated chronic complications. Modern antidiabetic therapies aim to improve overall metabolic health, focusing on both glucose homeostasis and broader systemic benefits [1]. Among these therapies, incretin-based treatments have gained considerable attention due to their innovative mechanism of mimicking or amplifying the effects of endogenous incretin hormones, such as glucagon-like peptide-1 (GLP-1) and glucose-dependent insulinotropic polypeptide (GIP). Both GLP-1 and GIP primarily function by augmenting postprandial insulin secretion, thereby contributing to improved glycemic control [2].

In addition to their glycemic benefits, clinical trials have identified other advantages of incretin-based therapies, including weight reduction and cardiovascular and renal protection in patients with and without T2D, underscoring their potential for broader clinical applications [3,4,5]. The two main classes of incretin-based therapeutics are GLP-1 receptor agonists (GLP-1RAs), which mimic GLP-1 activity, and dipeptidyl peptidase-4 inhibitors (DPP-4is), which prevent the degradation of endogenous incretins. Tirzepatide, a novel dual GLP-1 and GIP receptor agonist, has shown promise in managing diabetes and promoting weight loss [6,7].

The scope of these therapies extends beyond glycemic control. In particular, GLP-1RAs have now been approved for the treatment of obesity, including in pediatric populations. This marks a significant advancement in addressing the growing global obesity epidemic, offering a pharmacological option for weight management in younger individuals. Furthermore, evidence suggests that incretin-based therapies may serve as adjunct treatments in type 1 diabetes (T1D), especially for patients struggling with glycemic variability and obesity-related comorbidities. While these agents do not replace insulin therapy in T1D, they can enhance postprandial glucose regulation and contribute to weight loss, providing additional therapeutic benefits.

Current clinical practice guidelines for T2D management recommend GLP-1RAs and dual agonists over insulin as preferred injectable options, citing their superior glycohemoglobin reduction, lower risk of hypoglycemia, and cardiorenal protective effects. These therapies are particularly recommended for patients with poorly controlled diabetes, established cardiovascular disease (CVD), or chronic kidney disease [8].

Despite their established efficacy, concerns have been raised regarding the safety of incretin-based therapies, particularly GLP-1RAs, due to a potential association with pancreatic and thyroid cancers [9]. Although the mechanisms underlying these potential carcinogenic effects remain poorly understood, the widespread expression of GLP-1 and GIP receptors across various tissues suggests potential pleiotropic effects beyond the pancreas. It is also important to consider that obesity and T2D themselves increase the risk of malignancies, including pancreatic and thyroid cancers [10,11,12,13]. Mechanistically, incretin-based therapies may influence tumorigenesis and tumor progression [14,15].

There is an increasing need to understand the oncologic implications of incretin-based therapies. While some studies suggest protective effects against cancer progression, others raise concerns about potential risks, particularly regarding thyroid and pancreatic malignancies. A systematic assessment of available data is essential to clarify these conflicting findings.

For example, several meta-analyses have demonstrated a reduced incidence of obesity-related cancers, including colorectal and prostate cancers, among patients using GLP-1RAs compared to those on insulin therapy. A recent systematic review found that GLP-1RA use was associated with a lower risk of 10 out of 13 common obesity-related malignancies. However, other research indicates an elevated risk of thyroid and pancreatic cancers in patients receiving long-term GLP-1RA therapy. Notably, Wang et al. reported an increased risk of thyroid cancer, a finding supported by adverse event reports in pharmacovigilance databases [16]. Similarly, a meta-analysis of nine randomized controlled trials found no increased cancer risk with tirzepatide use [17].

As the availability and clinical utilization of incretin-based medications continues to grow, it is crucial to carefully assess whether their benefits outweigh the potential risks. Therefore, we conducted a comprehensive review of recently published studies indexed in the PubMed database to evaluate the potential relationship between incretin-based therapies and cancer risk. Specifically, we focused on evidence related to DPP-4is, GLP-1RAs, and dual GLP-1/GIP receptor agonists in relation to various malignancies. While previous systematic reviews have addressed the safety of incretin-based therapies, our aim is to offer an updated summary of the latest findings, with an emphasis on potential mechanisms and clinical implications. Additionally, we discussed emerging agents under development and highlighted priority issues to guide future research.

## 2. DPP-4 Inhibitors and Cancer

The role of dipeptidyl peptidase-4 (DPP-4) in cancer remains complex and controversial, as studies have reported both tumor-promoting and tumor-suppressing effects [18]. DPP-4, also known as CD26 or adenosine deaminase complexing protein 2 (EC 3.4.14.5), is a homodimeric type II transmembrane glycoprotein. Its structure comprises a short cytoplasmic segment (amino acids 1–6), a 22-amino-acid transmembrane domain, and a large extracellular domain (amino acids 29–766). The enzyme’s catalytic triad, located in the extracellular domain, consists of the residues Ser630, Asp708, and His740 in the human protein [19].

DPP-4 expression in cancer tissues and its levels in body fluids have been proposed as potential biomarkers for various malignancies [20], as shown in Table 1. However, its precise function in cancer biology is not fully understood. To investigate this, a large-scale study monitored patients over a five-year period after initiating treatment with DPP-4 inhibitors (DPP-4i), GLP-1 receptor agonists (GLP-1RA), or metformin. The study included 344,550 patients in the DPP-4i group, 112,000 in the GLP-1RA group, and 1,245,930 in the metformin group, with participants in the GLP-1RA group having a higher average body mass index (BMI). Cancer occurrence over the five-year period was 9.5% in the DPP-4i group, 8.7% in the GLP-1RA group, and 9.3% in the metformin group.

To assess cancer risk, odds ratios (ORs) were calculated after a six-month lag period, adjusting for variables such as sex, age, smoking status, alcohol consumption, hemoglobin A1c (HbA1c), and BMI. The adjusted odds ratios (aORs) for cancer incidence were 1.01 (95% CI: 0.94–1.08) for DPP-4i and 1.06 (95% CI: 0.93–1.20) for GLP-1RA, compared to metformin. These findings indicate no significant overall increase in cancer risk associated with either DPP-4i or GLP-1RA use.

However, subgroup analyses revealed cancer type-specific associations. DPP-4i use was linked to a higher risk of bladder, kidney, and liver, cancers, and melanoma, but a reduced risk of breast, lung, and prostate cancers. In contrast, GLP-1RA use was associated with an increased risk of thyroid cancer but a decreased risk of bladder, colon, lung, and prostate cancers [21].

The effects of DPP-4 inhibitors (DPP-4i) on postoperative outcomes and tumor biology in diabetic patients with colorectal cancer (CRC) were investigated in a retrospective study involving 260 patients with T2D who underwent curative resection for CRC. Among these, 135 were DPP-4i users, and 125 were non-users. The 5-year disease-free survival (DFS) rate was significantly lower in DPP-4i users compared to non-users (73.7% vs. 87.4%; HR, 1.98; 95% CI, 1.05–3.71; *p* = 0.035). Immunohistochemical analyses revealed distinct tumor microenvironment features in DPP-4i users, including increased Zeb1+ tumor cells, reduced CD3+ and CD8+ T cell infiltration, and fewer tertiary lymphoid structures (*p* < 0.001). Moreover, tumors from DPP-4i users exhibited a higher density of M2-type macrophages (CD68+ CD163+). These findings suggest that DPP-4i may facilitate the epithelial-to-mesenchymal transition (EMT) and foster a tumor-supportive immune microenvironment, potentially impairing CRC outcomes in T2D patients [22].

In prostate cancer, the impact of DPP-4i seems more favorable. A retrospective study found that prostate cancer patients treated with DPP-4i exhibited improved survival rates compared to non-users, independent of metformin use. This survival benefit was not observed in pancreatic or breast cancer patients, which may relate to the differential expression profiles of CD26/DPP-4. Immunohistochemistry data from the Protein Atlas indicate moderate to strong CD26/DPP-4 expression in prostate cancers, a characteristic absent in most other cancers, including pancreatic and breast cancers. Notably, CD26/DPP-4 activity is approximately twice as high in prostate cancer tissue as in benign prostatic tissue, potentially contributing to tumor growth. Despite this, high CD26/DPP-4 expression in prostate cancer is paradoxically associated with poor prognosis. These findings suggest that DPP-4 inhibition may enhance survival outcomes in prostate cancer, although further mechanistic studies are warranted [23].

DPP-4 inhibitors may also be promising in combination therapies for non-small cell lung cancer (NSCLC). While the PD-L1 blockade has shown significant efficacy in improving NSCLC survival, resistance remains a challenge due to tumor heterogeneity and individual immune differences. In preclinical studies, the DPP-4i anagliptin enhanced the tumor-suppressive effects of PD-L1 inhibitors when used in combination, suggesting a regulatory role for DPP-4i in the tumor microenvironment. This finding positions DPP-4i as a potential adjunct in tumor immunotherapy for NSCLC [24].

Tyrosine kinase inhibitors (TKIs) are a mainstay for treating advanced renal cell carcinoma (RCC), but resistance frequently develops. Cancer stemness, a driver of aggressiveness and treatment resistance, has been linked to DPP-4 activity. In RCC spheroids derived from patients, DPP-4 expression correlated with stemness-related genes. Both in vitro and in vivo studies showed that DPP-4 inhibition, via sitagliptin or targeted siRNA, reversed sunitinib resistance. Additionally, DPP-4 expression increased with retinoic acid exposure and decreased with ALDH1A inhibition. In T2D patients with DPP-4-high RCC tumors, treatment with DPP-4i improved TKI responses, suggesting DPP-4 inhibition could be a potential strategy to overcome TKI resistance [25].

Hepatocellular carcinoma (HCC) is characterized as an “immune-cold” tumor due to low T cell infiltration. First-line treatments for advanced HCC combine immune checkpoint inhibitors, which enhance T cell activation, with VEGF inhibitors, which promote T cell trafficking into tumors. CD26/DPP-4, a serine protease, cleaves chemokines critical for immune cell recruitment. This cleavage weakens anticancer immunity while exacerbating insulin resistance and inflammation, both of which are risk factors for HCC. Although DPP-4 inhibition may theoretically enhance T cell trafficking, further studies are required to determine its role in altering the immune landscape in HCC [26].

**Table 1:** Studies evaluating the relationship between DPP-4i and cancer risk.

Type of Cancer	Study	Method	Design	No. of Participants/ Samples	Result
Animal studies					
Liver	Sohji et al. [26]	In vitro	HCC cell lines, xenograft tumors injected in mice models	41	No antitumor effect in the in vitro models (*p* < 0.05)
Lung	Zuo et al. [24]	In vitro	Tissue sampling of LC cells injected mice, fed with DPP-IV	20	Enhancement of immunotherapy effect by inhibition of tumor-associated macrophage activity (*p* < 0.05)
Human Studies					
Pancreas	Shah et al. [23] Elashoff et al. Wang et al. [21]	Real world Real world Real world	Patients with PC and T2D Database analysis of AE Database analysis of AE	5.359 1863 5095	No significant effect on overall survival (HR 17 (95% CI: 0.93–1.24, *p* = 0.68) Higher risk of PC (*p* < 0.8) No significant effect on cancer risk (OR 0.94 (95% CI 0.86–14, *p* < 0.23))
Colorectal	Saito et al. [22]	Real world	Patients with CRC and T2D	260	Lower disease-free survival (HR 1.98 (95% CI 15–3.71, *p* = 0.35))
Liver	Wang et al. [21]	Real world	Database analysis of AE	344.550	Higher risk of liver cancer (OR 1.14 (95% CI 12–1.26, *p* = 0.2))
Kidney	Wang et al. [21] Kamada et al. [25]	Real world In vitro	Database analysis of adverse events PDTS of patients with RCC and T2D	344.550 769	Higher risk of kidney cancer (OR 1.13 (95% CI 14–1.23, *p* < 0.01)) Enhancement of tumor-suppressive TKI efficacy (*p* < 0.05)
Lung	Wang et al. [21]	Real world	Database analysis of AE	344.550	Reduced risk of LC (OR 0.91 (95% CI 0.86–0.97, *p* < 0.01))
Breast	Wang et al. [21] Shah et al. [23]	Real world Real world	Database analysis of AE Patients with BC and T2D	344.550 1685	Reduced risk of BC (OR 0.90 (95% CI 0.85–0.94, *p* < 0.01)) No significant effect on overall survival (HR 17 (95% CI: 0.93–1.25, *p* = 0.33)
Prostate	Wang et al. [21] Shah et al. [23]	Real world Real world	Database analysis of AE Patients with PRC and T2D	344.550 15.330	Reduced risk of PRC (OR 0.87 (95% CI 0.82–0.91, *p* < 0.01)) Improves overall survival (HR 0.77 (95% CI: 0.64–0.93), *p* = 0.5)
Bladder	Wang et al. [21]	Real world	Database analysis of AE	344.550	Higher risk of bladder cancer (OR 1.18 (95% CI 19–1.29, *p* < 0.01))

HCC—hepatocellular cancer; LC—lung cancer; PC—pancreatic cancer; T2D-type 2 diabetes, CRC—colorectal cancer; PDTS—patient-derived tissue sphenoids; RCC—renal cell cancer; BC—breast cancer; PCR—prostate cancer; HR—hazard ratio; OR—odds ratio; TKI—tirozin kinase inhibition; AE—adverse events; OR—odds ratio; HR—hazard ratio; CI—confidence interval.

DPP-4 plays a multifaceted role in cancer biology, including its involvement in cell differentiation, adhesion, immunomodulation, and apoptosis [27]. While DPP-4i do not appear to increase overall cancer incidence, they may influence tumor progression and metastasis in specific cancer types [28,29]. By regulating biopeptide activity, DPP-4 cleaves peptides, cytokines, and chemokines, which are essential to the tumor microenvironment [30]. In CXCR4-positive cancers, such as breast cancer, the CXCL12/CXCR4 axis drives metastasis to organs rich in CXCL12, including the lungs, bone marrow, and lymph nodes. DPP-4 inhibition may elevate CXCL12 levels, potentially promoting metastasis in these cancers [31,32].

## 3. GLP-1 Receptor Agonists and Cancer

Glucagon is a peptide hormone produced by pancreatic alpha cells, which are located in the pancreas where they are scattered in small clusters throughout the organ (islets of Langerhans). The precursor of glucagon is a protein called proglucagon, which comes from preproglucagon and is generated in the alpha cells of the pancreas; in the intestinal L cells (in the distal ileum and colon); and in certain neurons in the brain. Accordingly, the same proglucagon gene is processed differently in intestinal L cells and neurons, thereby producing GLP-1, glucagon-like peptide-2 (GLP-2), and glicentin. GLP-1 and GLP-2 induce glucose-stimulated insulin synthesis but have distinct roles, whereby GLP-1 is primarily involved in glucose regulation, i.e., it enhances insulin secretion; suppresses glucagon secretion; delays stomach emptying; and promotes feelings of satiety or fullness, and GLP-2 is primarily involved in maintaining and repairing the intestinal lining (i.e., it improves the absorption of nutrients; reduces gut permeability; and stimulates cell proliferation) [33]. The most significant effects of GLP-1 involve the regulation of blood glucose levels in individuals with T2D through promoting glucose-dependent insulin release, inhibiting glucagon production, stimulating beta cell growth, enhancing insulin secretion, and limiting weight gain.

On the other hand, concerns have arisen regarding the safety of GLP-1RA and the potential development of carcinomas. Studies suggest that compared to individuals with T2D receiving insulin, those who received GLP-1RAs had a significantly reduced risk of developing 10 of 13 common obesity-associated cancers, such as the following (from lowest to highest risk): gallbladder; meningioma; pancreatic; hepatocellular; ovarian; multiple myeloma; esophageal; endometrial; colorectal; and kidney cancer [9,34,35]. However, Wang and Kim have shown that GLP-1 is associated with a higher risk of certain malignancies compared to metformin [36]. They also reported that GLP-1RAs is associated with lower risk of prostate; lung; and colon cancer, but a higher risk of thyroid cancer, which aligned with findings from the FDA Adverse Event Reporting System (FAERS) database [36]. Most of the literature available concentrates on investigating the potential of GLP-1RA in carcinoma such as breast: hepatocellular: pancreatic: kidney: thyroid: prostatic: and colorectal carcinoma.

One study found that obese individuals with T2D using GLP-1RAs had a reduced risk of breast cancer or benign neoplasms (Table 2). GLP-1 expression has been observed in breast cancer, where it influences not only the increase in cAMP levels and activation of CREB, but also the activation of GLP-1 and the inhibition of NF-kβ activation, leading to inhibited proliferation of breast cancer cells [37,38,39,40]. Conversely, the results of a randomized trial that followed 14,752 patients for a period of 3.2 years following subcutaneous administration of GLP-1RA did not show that a reduced risk of breast cancer exists [40].

The findings of animal studies performed on nonalcoholic steatohepatitis (NASH) and nonalcoholic fatty liver disease (NAFLD) in individuals which developed HCC have significantly expanded our knowledge of both basic and molecular processes which lead to the development of this malignancy. The results of these studies also indicated that pharmaceutical agents such as GLP-1RA may participate in the molecular pathways responsible for the initiation and progression of HCC [16,41]. One clinical trial that included individuals suffering from NASH showed that neoplasms (including cysts and polyps) occurred slightly more often in the GLP-1RA group as opposed to the control one [42].

The outcomes of studies investigating pancreatic cancer demonstrated the antitumor effects of GLP-1 on gemcitabine-resistant pancreatic cancer cells, and suggest that GLP-1 agonists, via the modulation of the NK-kβ signaling pathway, enhance the therapeutic effect of gemcitabine [43]. However, various studies have produced contradictory results concerning the development of pancreatic cancer; namely, some suggest the development of pancreatic cancer in T2D individuals who are treated with GLP-1RA, as opposed to those who are treated with insulin. These findings are explained by the fact that long-term use of GLP-1RAs is associated with the development of pancreatitis, which is a risk factor for pancreatic cancer. The findings of studies performed on experimental rats have shown that GLP-1RAs can not only promote pancreatic inflammation and increase serum lipase levels, but also induce columnar cell atypia, resembling a precursor lesion of cancer [43,44,45]. On the other hand, other studies, such as one population-based cohort study, showed reduced incidence of pancreatic cancer in T2D individuals that were treated with GLP-1RA [9]. In their 15-year follow-up study, Wang et al. observed a significantly lower incidence of pancreatic cancer in T2D individuals treated with GLP-1RA compared to insulin [16].

In the same study, Wang et al. reported that GLP-1RAs exert direct effects on renal function unrelated to mitogenesis. GLP-1RA was associated with an increased risk of kidney cancer as compared to metformin, whereas it is reduced compared to insulin [16,46].

Findings of animal studies have shown that thyroid C cells of neoplastic lesions express GLP-1. The implementation of GLP1-RA leads to the activation of GLP-1R in thyroid C cells, the promotion of their hyperplasia, and an increase in calcitonin synthesis, which increases the risk of the development of medullary thyroid cancer [47,48,49]. In their study, Wang et al. suggested that the implementation of GLP-1RA may be associated with increased risk of thyroid cancer, which is supported by findings from the French National Health Data System, thus indicating that if it is implemented for one to three years, it carries an increased risk of developing all thyroid gland cancers [16,50]. Together, these studies support the view that GLP-1RA should not be used in patients with multiple endocrine neoplasia syndrome type 2 (MEN 2) nor those with a positive familial history.

**Table 2:** Studies’ evaluation of the relationship between GLP1RA and cancer risk.

Type of Cancer	Study	Method	Design	No. of Participants	Result
Animal studies					
Thyroid	Madsen et al. [48]	In vitro	Tissue sampling of GLP1RAinjected mice	10	Increased incidence of C cell hyperplasia and calcitonin levels in GLP1R wild type mice (*p* < 0.01)
Human studies					
Thyroid	Sun et al. [35] Wang et al. [37] Silverii et al. [50]	RCT RCT RCT	Database genetic analysis Database analysis of AE Database analysis	65.328 64.230 46.228	No increased risk of thyroid cancer (OR 0.83 (95% CI 0.63–1.10, *p* = 0.187)) Increased risk of thyroid cancer (aOR 1.65, *p* < 0.05) Increased overall risk of thyroid cancer (OR 1.52 (95% CI 11–2.29, *p* = 0.4))
Pancreas	Sun et al. [35] Dankner et al. [45]	RCT Real world	RCTs Prospective study	65.328 33.370	No increased risk of PC (OR 0.78 (95% CI 0.61–1, *p* = 0.19)) No increased risk of PC (HR 0.50 (95% CI, 0.15–1.71, *p* > 0.05))
Colorectal	Sun et al. [35] Wang et al. [37]	RCT RCT	Database genetic analysis Database analysis of AE	65.328 64.230	Increased risk of CRC (OR 1.12 (95% CI 17–1.18, *p* < 0.01)) Reduced risk of CRC (0.85, *p* < 0.3)
Breast	Sun et al. [35] Wang et al. [21]	RCT RCT	Database genetic analysis Database analysis of AE	65.328 14.752	Reduced risk of BC (OR 0.92 (95% CI 0.88–0.96, *p* < 0.01)) No increased risk of BC (OR 0.99 (95% CI 0.91–17, *p* = 0.75))
Lung	Sun et al. [35] Wang et al. [37]	RCT RCT	Database genetic analysis Database analysis of AE	65.328 64.230	No increased risk of LC (OR 11 (95% CI 0.93–1.10, *p* = 0.76)) Reduced risk of LC (aOR 0.81, *p* = 0.5)
Liver	Wang et al. [43]	Real world	Database analysis	22.575	Reduced risk of liver cancer (HR 0.63 (95% CI 0.26–1.50, *p* < 0.05))
Prostate	Sun et al. [35] Wang et al. [37]	RCT RCT	Database genetic analysis Database analysis of AE	65.328 64.230	Increased risk of PRC (OR 19 (95% CI 15–1.14, *p* < 0.01)) Reduced risk of PRC (aOR 0.72, *p* = 0.8)

RCT—randomized control trial, AE—adverse event; PC—pancreatic cancer; CRC—colorectal cancer; BC—breast cancer; LC—lung cancer; PCR—prostate cancer; OR—odds ratio; HR—hazard ratio; CI—confidence interval.

Increased expression of GLP-1R was found in both normal colon tissue and in colorectal cancer tissue, which is probably due to DNA hypermethylation [51]. In addition, GLP-1RA can activate the Wnt signaling pathway, which also plays a significant role in the development of colorectal cancer [52]. The results of one retrospective study showed an association between GLP-1RA and reduced risk of colorectal cancer [53].

Animal studies have also revealed that in prostate cancer, GLP-1 analogs activate the p38 signaling pathway in LNCap cells without affecting the ERK1/2 pathway, thereby suppressing LNCap cell proliferation and inducing apoptosis [54,55,56]. Moreover, samples of human prostate cancer tissue and cell lines show an expression of GLP-1R, which may help decrease prostate cancer growth by inhibiting the activation of the ERK-MAPK signaling pathway [57].

Summarizing potential cancer risk reduction, liraglutide, semaglutide, and dulaglutide have been linked to lower incidences of colorectal and prostate cancer. Exenatide and liraglutide may reduce tumor growth, though large trials show no clear risk reduction for breast cancer. Semaglutide may lower incidence for pancreatic cancer. Semaglutide and dulaglutide were associated with a lower risk of lung and kidney cancer.

Nonetheless, there are possible carcinogenic risks that should be emphasized. Liraglutide and semaglutide may increase C cell hyperplasia and the risk of medullary thyroid carcinoma. Exenatide and liraglutide are linked to higher rates of pancreatitis, a risk factor for malignancy. Liraglutide users have demonstrated a higher risk of kidney cancer compared to metformin users. Overall, the evidence is mixed; while GLP-1RAs may lower the risk of some cancers, they could increase susceptibility to others. Further long-term studies and patient-specific risk assessments are necessary. In conclusion, GLP-1 and its receptor agonists have been studied for their potential in various types of carcinomas. However, to fully explore and elucidate their exact role in different developmental molecular pathways, additional, broader, and more specialized studies are required.

## 4. Dual GIP and GLP-1 Receptor Agonists and Cancer

In the context of the obesity pandemic and its role in type 2 diabetes (T2D) pathophysiology, there is a pressing need for therapies targeting multiple metabolic pathways, particularly those involved in energy metabolism and appetite regulation [58]. The structural similarity of glucagon-like peptide-1 (GLP-1) and glucose-dependent insulinotropic polypeptide (GIP), along with their selective receptor binding properties, has led to the development of dual agonists capable of synergistically enhancing insulin secretion.

Tirzepatide, a dual GLP-1/GIP receptor agonist (GLP-1/GIP-RA), holds a prominent place in the T2D treatment algorithm. It demonstrates greater affinity for GIP receptors (GIP-R) than GLP-1 receptors (GLP-1R), and has been shown to improve glycemic control, reduce body weight, and confer cardiovascular benefits. Additionally, GIP may promote β cell regeneration by reducing apoptosis and increasing β cell mass. In patients with normoglycemia, GIP stimulates glucagon secretion, but this effect is altered in T2D, thus preserving its positive impact on glucagon regulation [59].

Active forms of GLP-1 and GIP also influence atherosclerosis and macrophage foam cell formation by activating cAMP and downregulating CD36 and ACAT-1, suggesting potential anticancer effects [60]. However, concerns remain regarding the possible promotion of cancers in the pancreas, thyroid, breast, liver, and colon [17].

The effects of tirzepatide on carcinogenesis are mediated through GLP-1R, GIP-R, and the inhibition of dipeptidyl peptidase-4 (DPP-4). Pancreatitis, a known risk factor for pancreatic cancer, has been observed with GLP-1RAs, and long-term malignant transformation of normal pancreatic duct cells remains a concern. Estimates suggest a 20-year latency period before pancreatic ductal cells develop malignancy and metastasis [61]. Similarly, tirzepatide is contraindicated in individuals with a personal or family history of medullary thyroid carcinoma (MTC) or multiple endocrine neoplasia type 2 (MEN2) due to its association with medullary thyroid cancer in both preclinical and human studies [62,63,64].

GLP-1R is overexpressed in certain benign and malignant endocrine tumors, but is generally absent in carcinomas, except for low levels in ovarian and prostate carcinomas. In rats, medullary thyroid carcinomas express GLP-1Rs at nearly eight times the levels found in humans [65]. In a study by Regazzo et al., GIP-R expression in MTC specimens was correlated with tumor progression markers, including larger tumor size, advanced stage, and higher Ki-67 levels, indicating its role as an index of malignancy [66].

Tirzepatide’s effects on other cancers are less well understood. While GIP-R is expressed in various tissues—including the pancreas, bones, brain, and adipose tissue—its role in carcinogenesis remains under investigation. GIP has been implicated in processes such as cell proliferation and differentiation, which may stimulate the growth of certain cancers [67,68]. For example, colorectal cancer cells express GIP-R, and its association with obesity may contribute to a higher incidence of colorectal carcinoma in this population. However, evidence from randomized controlled trials indicates that tirzepatide does not significantly affect cancer risk overall [17].

## 5. Discussion

### Balancing Risks and Benefits

Despite concerns about pro-oncogenic effects, dual GIP/GLP-1RAs offer significant potential for mitigating cancer risks in patients with metabolic syndrome. By reducing glucose levels, body weight, and inflammation, these agents may inhibit cancer growth and progression, which is particularly relevant given the higher cancer incidence associated with obesity and diabetes.

In parallel, GLP-1 receptor agonists (GLP-1RAs) such as liraglutide, semaglutide, and exenatide have demonstrated promising effects on tumor biology. Preclinical studies suggest that GLP-1RAs can modulate key signaling pathways—such as MAPK and PI3K/AKT—to reduce cellular proliferation and induce apoptosis in certain cancer types. However, clinical findings remain mixed; while some data indicate a reduced risk for colorectal and prostate cancers, other reports have raised concerns about increased risks of thyroid and pancreatic malignancies.

The role of incretin-based therapies in cancer risk remains a topic of ongoing debate. Current findings suggest a nuanced relationship, with some studies highlighting protective effects, while others indicate potential oncogenic risks. This inconsistency underscores the complexity of incretin hormone signaling in different cancer types.

DPP-4 inhibitors (DPP-4is) also present a dichotomy in cancer outcomes. While some studies suggest that DPP-4is may promote the epithelial-to-mesenchymal transition (EMT), a hallmark of cancer progression, others indicate a survival advantage in prostate cancer patients. The expression of CD26/DPP-4 in different tissues may account for these conflicting results. Research has shown that prostate cancer cells exhibit elevated DPP-4 activity, potentially explaining the survival benefits observed in DPP-4i users. However, higher risks of bladder, kidney, and liver cancers have been noted, emphasizing the need for further long-term observational studies.

Preclinical models have suggested protective effects in various tissues, including the brain, pancreas, intestine, kidneys, liver, heart, and vascular endothelium. These effects may stem from alterations in insulin signaling and reductions in systemic inflammation. However, long-term studies are needed to confirm these findings and establish their safety profile in cancer patients [69].

## 6. Clinical Implications

Tirzepatide and other dual GLP-1/GIP receptor agonists represent a promising step forward in the treatment of metabolic disorders. However, their intersection with cancer biology demands careful consideration. While initial data suggest a generally safe profile, specific risks related to pancreatic and thyroid cancers, particularly in genetically predisposed individuals, require further investigation. Future research should focus on refining the understanding of these dual agonists’ pharmacological profiles, balancing their metabolic benefits against potential oncogenic risks.

The diverging effects of incretin-based therapies on cancer risk suggest that a patient-specific approach is necessary. Clinicians should weigh the metabolic benefits of these therapies against their potential oncogenic risks, especially in individuals with a personal or family history of thyroid or pancreatic cancers. Moreover, patient education regarding potential adverse effects and the importance of regular cancer screenings is critical.

Pharmacovigilance data also highlight the necessity of post-marketing surveillance. Regulatory agencies, such as the FDA and EMA, continue to assess emerging data to refine safety guidelines. Future updates to clinical practice recommendations may incorporate risk stratification models that help identify the patients most likely to benefit from incretin-based therapies with minimal cancer risk.

## 7. New Agents Under Development

### 7.1. Emerging Therapies and Their Safety Profiles

Other entero-pancreatic hormones, such as GIP, glucagon, and amylin, are currently undergoing clinical trials. Although the relatively short duration of randomized controlled trials (RCTs) often precludes detecting malignancies, preliminary data suggest that the safety profiles of these agents may be comparable to those of established treatments (Table 3).

**Orforglipron**: A novel, non-peptide GLP-1 receptor agonist (GLP-1RA) developed for oral administration, orforglipron has shown promise in managing diabetes and obesity. A recent meta-analysis of four RCTs involving 744 participants reported that the most common adverse events were gastrointestinal, primarily at higher doses. Notably, its effects on pancreatic enzymes and calcitonin levels were consistent with those observed in other GLP-1RA classes [70,71,72]. Similar findings were reported in another meta-analysis by Karakasis et al., which included data from seven RCTs involving 1037 patients treated with orforglipron or danuglipron, another oral GLP-1RA [73].

**Retatrutide**: A triple receptor agonist targeting GLP-1, GIP, and glucagon receptors, retatrutide has demonstrated safety in phase II trials involving patients with obesity and metabolic dysfunction-associated steatotic liver disease (MASLD). Importantly, these studies did not identify cases of medullary thyroid cancer or C cell hyperplasia [74,75].

**Cagrilintide**: An amylin analog currently under evaluation in combination with semaglutide as *cagrisema*, cagrilintide has shown encouraging safety and efficacy profiles in phase I and II trials for obesity and T2D [76,77].

**Table 3:** Studies evaluating the relationship between dual (GLP1R and GIP-R) agonists and cancer risk.

Type of Cancer	Study	Method	Design	No. of Participants	Result
Animal studies					
Colorectal	Prabakaran et al. [74]	In vitro	PCR analysis of GIP-R in mice CRC cell lines	/	Increased expression of GIP-R in colorectal cancer and dose-dependent CRC cell proliferation (*p* < 0.01)
Thyroid	Waser et al. [70]	In vitro	PCR analysis of GLP-1R and GIP-R expression in MTC cells	/	Increased expression of GIP-R in MTC in rodents
Human studies					
Thyroid	Regazzo et al. [71] Popovic et al. [17]	In vitro RCTs	PCR analysis of GIP-R in tumor specimensDatabase analysis of AE	49 978	GIP-R expression in 80% of patients with MTC (*p* < 0.05) No increased risk of thyroid cancer (RR 0.35 (95% CI 0.13–0.95, *p* = 0.61))
Colorectal	Prabakaran et al. [74]	In vitro	PCR analysis of GIP-R in human CRC cell lines	/	Increased expression of GIP-R and dose-dependent CRC cell proliferation (*p* < 0.01)
Breast	Samuel et al. [75]	In vitro	PCR analysis of GIP-R expression in cancer lines	8.401	Aberrations in GIP signaling associated with increased risk of BC

PCR—polymerase chain reaction; MTC—medullary thyroid cancer; CRC—colorectal cancer; AE—adverse events; BC—breast cancer; AE—adverse events; RR—risk ratio; CI—confidence interval.

Ongoing phase III trials for agents such as survodutide, retatrutide, and cagrisema aim to provide a more comprehensive understanding of their safety and efficacy in individuals with T2D and/or obesity. These longer trials will help address the limitations of shorter phase II studies, particularly regarding rare adverse events, including malignancy risks [78].

### 7.2. Anticancer Potential of Novel Agents

Preliminary evidence suggests that certain novel therapies may exhibit anticancer effects in addition to their metabolic benefits:

**Survodutide**: A dual agonist of glucagon and GLP-1 receptors, survodutide has shown potential benefits in metabolic dysfunction-associated steatohepatitis (MASH), including improving fatty acid oxidation, reducing lipogenesis, and suppressing hepatic inflammation. Phase II trials reported no new safety concerns and highlighted its potential to slow the progression of cirrhosis to hepatocellular carcinoma (HCC) [79,80,81].

**Pemvidutide**: Another promising agent in this class, pemvidutide demonstrated favorable effects in overweight and obese subjects with MASLD during a 12-week randomized, double-blind, placebo-controlled clinical trial. These findings support further evaluation of its role in MASH and its potential implications for cancer prevention [82].

While these findings are promising, further research is warranted to clarify the mechanisms by which these therapies might impact cancer risk and progression.

## 8. Ongoing Challenges and Regulatory Oversight

Although incretin-based therapies generally have favorable safety profiles with respect to cancer risk, the possibility of rare adverse events such as malignancies cannot be completely excluded. Regulatory agencies, including the U.S. Food and Drug Administration (FDA) and the European Medicines Agency (EMA), continuously monitor emerging data, update safety information, and refine guidelines based on results from ongoing research, post-marketing surveillance, and adverse event reporting.

## 9. Considerations for Clinical Practice

A thorough risk–benefit analysis is crucial when prescribing incretin-based therapies, balancing the advantages of improved glycemic control and weight loss against potential cancer risks. Patient-specific factors—such as age, duration of diabetes, comorbidities, personal and family history of cancer, genetic predisposition, and relevant biomarkers—should be carefully evaluated to guide therapy selection. Patient education plays an equally important role; informing patients about potential risks and benefits helps them make well-informed decisions and emphasizes the importance of regular participation in cancer screening programs. Ongoing monitoring through routine follow-ups is essential for assessing side effects and ensuring early detection of any malignancies, thereby maximizing the therapeutic benefits while minimizing potential harms.

## 10. Future Directions

To optimize the use of incretin-based therapies, future research should focus on long-term cancer risk assessment by extending follow-up durations in clinical trials, which will allow for a more comprehensive evaluation of malignancy risks. Mechanistic studies are also needed to elucidate the biological pathways through which these therapies might influence tumor development or suppression. Moreover, personalized medicine approaches—such as identifying patient-specific factors that affect cancer risk—could enable clinicians to tailor treatment strategies and maximize therapeutic benefits while minimizing potential adverse outcomes.

## 11. Conclusions

Current evidence from clinical trials and real-world studies indicates that incretin-based therapies generally have a favorable safety profile regarding cancer risk. GLP-1 receptor agonists (GLP-1RAs) have been extensively studied for their potential role in various carcinomas, yet their precise impact on the molecular pathways involved in cancer development and progression remains unclear. Broader, more targeted research is needed to elucidate these mechanisms and address unresolved questions.

Dual GIP/GLP-1 receptor agonists offer promising therapeutic options for obesity and metabolic disorders. However, their intersection with cancer biology requires ongoing scrutiny, particularly given the complex interactions between metabolic regulation, tumorigenesis, and individual patient factors. Comprehensive evaluation of these agents’ safety and efficacy in cancer patients is critical as our understanding of their pharmacological effects continues to evolve.

The strengths of the study include a comprehensive evaluation of the relationship between incretin-based therapies and cancer risk, incorporating both human and animal studies, offering a broad perspective on the topic, and addressing both potential protective and adverse effects, ensuring a balanced analysis and integrating the latest research findings, providing up-to-date insights. However, there are some limitations. Some findings rely on observational data, which may introduce biases. The lack of long-term studies limits the ability to determine the prolonged effects of these therapies on cancer development. Furthermore, differences in study design and patient populations make direct comparisons challenging, and potential confounding factors, such as obesity and metabolic syndrome, complicate result interpretation. Therefore, further systematic analyses are needed.

Future research should prioritize long-term studies, gain mechanistic insights, and develop personalized risk assessments to ensure the safe and effective integration of incretin-based therapies into clinical practice, particularly for individuals at higher risk of cancer.

## Data Availability

No new data were created or analyzed in this study.
